# Co-Infection Rates between SARS-CoV-2 and RSV in Oropharyngeal, Nasopharyngeal Aspirate and Saliva Samples of COVID-19 Patients, Shiraz, South of Iran

**DOI:** 10.30476/DENTJODS.2022.92797.1679

**Published:** 2023-06-01

**Authors:** Jannan Ghapanchi, Ali Dehghani Naghvani, Fahimeh Rezazadeh, Mitra Farzin, Afagh Moatari, Sina Masoudi, Mohamadreza Kalantari, Reza Derafshi, Hossein Sedarat

**Affiliations:** 1 Dept. of Oral and Maxillofacial Medicine, School of Dentistry, Shiraz University of Medical Sciences, Shiraz, Iran; 2 Dept. of Oral Pathology, Biomaterials Research Center, School of Dentistry, Shiraz University of Medical Sciences, Shiraz, Iran; 3 Oral and Dental Disease Research Center, Dept. of Oral and Maxillofacial Medicine, School of Dentistry, Shiraz University of Medical Sciences, Shiraz, Iran; 4 Dept. of Prosthodontics, Biomaterials Research Center, School of Dentistry, Shiraz University of Medical Sciences, Shiraz, Iran; 5 Dept. of Microbiology, School of Dentistry, Shiraz University of Medical Sciences, Shiraz, Iran; 6 Undergraduate Student, Student Research Committee, School of Dentistry, Shiraz University of Medical Sciences, Shiraz, Iran; 7 Medical Student, Student Research Committee, Jahrom University of Medical Sciences, Jahrom, Iran

**Keywords:** Oral, Pharynx, Nasal, Aspirate, RSV, SARS-COV-2, Saliva

## Abstract

**Statement of the Problem:**

Determining the prevalence of respiratory viruses' coinfection with coronavirus disease 2019 (COVID-19) is essential to defining its true clinical influence.

**Purpose:**

This study aimed to evaluate co-infection rates between severe acute respiratory syndrome–related coronavirus 2 (SARS-CoV-2) and respiratory syncytial virus (RSV) in infected patients in Shiraz, south of Iran.

**Materials and Method:**

In a cross-sectional descriptive study, oropharyngeal, nasopharyngeal aspirate (NPA), and saliva samples of 50 COVID-19 patients who were referred to Ali-Asghar hospital (Shiraz, Iran) from March to August 2020, were collected. A control group consisted of age and sex-matched healthy participants. The nasopharyngeal and oropharyngeal aspirates were collected by sterile swabs. All cases were hospitalized, and all SARS-CoV-2 patients had a fever and respiratory symptoms. The samples were packed in a vial with 1 mL of transport medium and transported to the Valfagre specialty laboratory, where they were tested for RSV using a real-time polymerase chain reaction (PCR).

**Results:**

100 nasopharyngeal/oropharyngeal aspirates and saliva samples including 50 healthy controls (24 females, 26 males) and 50 COVID-19 patients' samples (27 males and 23 females) were studied. There was no significant difference regarding age as well as gender between both groups (*p*> 0.05). None of the healthy subjects was infected with RSV; however, 5(10%) patients from COVID-19 group were infected with the RSV virus. Chi-square test did not show a significant difference between RSV infection in COVID-19 patients and healthy subjects.

**Conclusion:**

The outcome of present research showed that concurrent RSV with COVID 19 infection might be seen in hospitalized patients in Shiraz Southwest of Iran. For more reliable findings, further research on bigger populations, including more pathogens in several places around the country, and considering the severity of symptoms is required.

## Introduction

Since December 2019, coronavirus disease 2019 (COV-ID-19) virulent disease has spread worldwide. Asia has had the majority of the reported cases of COVID-19 followed by Europe, North America, and Australia. Most of them were older than 18 years of age, and men consisted of 61.9% of the reported cases. Early patients occurred mostly amongst tourists from China and those who have had connection with them. Even Now, the constant local spread has led to the early epidemics in all states around the world [ 1 - 2 ]. COVID -19 cases first reported in the Persian Gulf region were linked to people who went from Iran and Iraq to Bahrain and Kuwait. COVID-19 is presently being fought with all of Iran's might, but control of the sickness has become very difficult concerning the infection's spread across the nation [ 3 ]. The human respiratory syncytial virus (RSV) is a pneumovirus belonging to paramyxoviridae group. This negative-sense single-stranded RNA virus is one of the highly widespread viruses to infect children globally and progressively is identified as a critical pathogen in adults, particularly the old subjects. Upper respiratory symptoms are the most common clinical signs in this illness. RSV commonly affects children, causing bronchiolitis, a lower respiratory tract infection with moderate breathing problems, and may rarely progress to pneumonia, respiratory failure, apnea, and decease [ 4 ]. Viral detection methods have significantly advanced in current years, as using molecular diagnostic techniques have considerably improved the capability to detect viruses [ 5 ].

The association between contamination with several respiratory viruses and the seriousness of the disease has not been well recognized. In studies that examined respiratory diseases by nucleic acid amplification techniques evaluating a significant number of viruses, such frequency was greater than 40% [ 6 - 7 ]. A study in Italy showed that coinfection of RSV and metapneumovirus was shortened the hospitalization and hypoxia, in comparison to RSV infection alone [ 8 ]. A French research similarly observed shorter duration of hospitalization in newborns with coexistent RSV and rhinovirus infection contrasting to single RSV infection [ 9 ].

 On the other hand, respiratory viral diseases have long been established as major causes of death and disability in adults and elders. The precise method by which these pathogenic agents, which ordinarily produce self-limited illnesses in healthy young individuals, are connected to severe aging symptoms is unknown [ 10 ]. Blunted immune response accompanied by aging-related physiologic changes may play a role in this problem. Influenza virus has usually got the greatest consideration followed by RSV and parainfluenza viruses that have been associated with serious illness in old subjects [ 10 ]. Determining the prevalence of coinfection of respiratory viruses with COVID-19 is essential to defining its true clinical influence. According to early reports from China, co-infection with other respiratory viruses was uncommon [ 11 ]. A research in the United States looked at coinfection in adults with COVID–19 and found that rhinovirus/enterovirus was the most prevalent (6.9%), followed by RSV (5.2%) [ 12 ]. Since the presence of more respiratory infections may increase the risk of morbidity among patients infected by COVID-19, well understanding of coinfection is critical. Hence, we report co-infection rates between SARS-CoV-2 and RSV in Shiraz, south of Iran.

## Materials and Method

### Ethical Statement

This cross-sectional study was carried out based on the guidelines of the *Declaration of Helsinki* as revised in Edinburgh (1975). The study protocol was approved by the Ethics Committee of Shiraz University of Medical Sciences, Shiraz, Iran encoded IR.SUMS.DENTAL. REC.1399.212. The written informed consents were obtained from participants for sample collection, and in unable cases, verbal consent was obtained. Patients were informed about the nature of study.

### Participants

In a cross-sectional descriptive study, oropharyngeal, nasopharyngeal aspirate (NPA), and saliva samples of 50 COVID-19 patients referred to Ali-Asghar hospital (Shiraz, Iran) from March to mid-August 2020, were collected. A control group of age and sex-matched healthy individuals was admitted to Oral Medicine Department of Shiraz School of Dentistry (IRAN) for normal exams at the same times as the research group. The exclusion criteria included patients with diabetes, pregnancy, smoking, using antibiotics (systemic or mouthwash). The samples were collected from the healthy control group and COVID-19 patients by convenient sampling. The nasopharyngeal and oropharyngeal aspirates were collected by sterile swabs. All cases were hospitalized and all SARS-CoV-2 patients had fever and respiratory symptoms (sore throat, cough, headache, and pain).They had positive polymerase chain reaction (PCR) test for COVID-19.

Whole-mouth saliva, parotid saliva, buccal, and palatal exfoliates were collected and processed for RSV amplification. The volunteers were asked to sit in a comfortable position and were asked to rinse their mouths with bottled water to remove food debris.

A trained nurse and a final-year dental student collected the throat swabs, NPAs, and saliva samples by using standard operating methods from patients who agreed to participate in the study. Demographic and clinical information were collected from each patient using a standardized data form.

The specimens were placed into a vial containing 1 mL of transport media that composed of phosphate buffer saline, glycerol, anti-biotic and anti-fungal. They were transported to the Valfagre specialty laboratory, where they were tested by using real-time PCR for RSV. The samples were divided into aliquots and stored at -80°C until use.

### Viral RNA detection

Viral nucleic acid was extracted from 0.2 mL of each respiratory specimen using the high pure viral nucleic acid extraction kit (Roche Diagnostics, Mannheim, Germany). Complementary DNA (cDNA) was synthesized using the first strand cDNA synthesis kit (Thermo Scientific, Waltham, MA USA). RNA integrity was confirmed by amplifying a genomic β-globin sequence as a reference gene.

We developed a highly sensitive and specific PCR using TaqMan technology, virus-specific primers, and probes designed to detect RSV (Table 1). In brief, 2µL of cDNA, 12.5µL of TaqMan Universal PCR Master Mix 0.8 µmol of each primer, and 0.4 µmol of the respective probe were combined and the volume adjusted to 20 µL with DNase-free water. The PCR reactions were performed with an ABI 7500 PRISM PCR system based on manufacturer’s protocol (Applied Biosystems, Waltham, MA, USA). Thermal cycling was initiated with uracil-Nglycosylase incubation at 50°C for 2 min and polymerase activation at 95°C for 10 min, followed by 45 cycles of 95°C for 15sec and annealing at 60°C for 60 seconds.

**Table 1:** Sequences of primers and probes designed for the detection of RSV

Virus	Primer	Sequence (5' to 3')	Length (bp)
RSV *	Forward	gtaacagaattgcagttgctcatg	
	Reverse	cgattgcagatccaacacctaac	178
	Probe	FAM **-cacaccagcagccaaca	
		atcgagcca-BHQ1 ***	

* RSV: Respiratory syncytial virus,

** FAM: 6-carboxyfluorescein

*** BHQ1: Black hole quencher 1

The threshold of recognition for RSV real-time PCR was 100 copies per reaction. The reaction was carried out in a final volume of 25µL containing 1 pmol of each primer, 2.5µL of 10× PCR buffer, 0.2mM of deoxynucleotide triphosphates (sNTPs), 2mM of MgCl_2_, 0.4µL of *Taq* DNA polymerase (CinnaGen, Tehran, Iran), and 2µL of cDNA. The amplification was started with an initial denaturation step at 95°C for 5 min, followed by 40 cycles of denaturation at 95°C for 45 sec, annealing at 55°C for 45 sec, an extension step at 72°C for 45 sec, and final extension at 72°C for 5 min, yielding a 169 base-pair (bp) product. The PCR products were separated using gel electrophoresis on a 1% agarose gel [ 13 ].

### Statistical Analysis

PSS software version 15 was used to conduct the statistical analysis for the case-control groups. To compare the positive and negative case instances in terms of age and gender status, the chi-square test was performed. Fisher's exact test was used to correlate the presence of RSV in both groups. Statistically, a significant difference was considered when *p* < 0.05.

### Results

**Figure 1:**
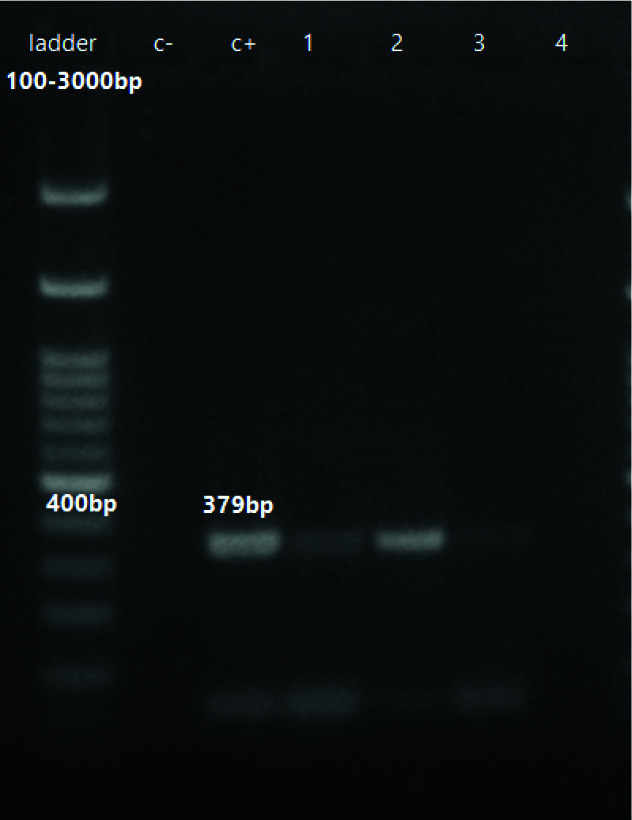
RSV detection on gel electrophoresis by PCR, 100 bp ladder, C +( positive control), C- (negative control), row 1 -3 positive samples

I100 nasopharyngeal/oropharyngeal aspirate, and saliva samples, including 50 healthy controls (24 females, 26 males) and 50 COVID-19 patients’ samples (27 males and 23 females) were studied. The age range was 15-59 years. The mean age of females and males was 35±13.4 and 33±15.2, respectively (Table 2). There was no significant difference regarding age as well as gender between both groups (*p*> 0.05). None of the healthy subjects were infected with RSV; however, 5 cases (10%) of the COVID-19 patients were infected with the RSV virus (Table 3). The polymerase chain reaction product on gel electrophoresis to detect RSV produced a band of about 379 bp (Figure 1). The chi-square test did not show a significant difference between RSV infection in COVID -19 patients and healthy subjects (*p*= 0.056).

**Table 2:** Sex distribution in patients and control groups

Groups (N)	Gender	Frequency	Percent %
Patients	Female	23	46%
	Male	27	54%
Control	Female	24	48%
	Male	26	52%

**Table 3:** PCR result of RSV in patients and control groups

	RSV*	
	Positive	Negative
Patients	5	45
Control	0	50
*p* Value	0.056	

* RSV: Respiratory syncytial virus

## Discussion

Studies showed that concurrent viral infection of the respiratory system with two viruses or more is not very usual in hospitalized cases, while it is not apparent whether these diseases are more or less serious than specific virus infections[ 14 - 15 ].

A Salivary, nasopharyngeal, and oropharyngeal aspirate samples from patients with COVID 19were examined for RSV. Present research, as the first study in this field in Iran, indicates a higher (10%) but non-significant RSV infection in patients with COVID-19 than healthy subjects. Considering the limited available data regarding co- or super-infection in COVID-19, our comparisons were limited. Iran has more victims in terms of this pandemic, so knowing all aspects of this disease is crucial. 

COVID-19 and RSV infections are individually infectious respiratory virus, but they are produced by two distinct viruses. Dual infection of RSV and SARS-CoV-2 may have a considerable consequence on the management and prognosis of the disease. Superimposition of viruses may lead to intensive care, higher doses of medication, long lasting disease, and susceptibility to ARDS. Additionally, coinfection may lead to severe immune deficiency. Correct and complete diagnosis of SARS-CoV-2 has a major role in prevention of the spread of the disease and management of corona and RSV.

Zandi *et al* [ 16 ] reported that in cases with sever COVID-19; the higher rate of coinfection especially with RSV could be seen than in mild forms. So prevention of the contamination may influence the prognosis especially in children. These findings differ with us due to our sample size and age [ 16 ].

Calvo *et al*. [ 15 ] compared simultaneous coinfection of RSV and rhinovirus in hospitalized children. Elevated fever degrees and hypoxia were more frequent in the RSV- rhinovirus coinfected cases than in specific virus infections. The adult and young adult patients with COVID-19 were compared with healthy subjects [ 15 ]. 

Goka *et al*. [ 17 ] did not find any finding to indicate the symptoms varied obviously between coinfection compared to single viral infections. Because the various characteristics of the virus may produce a bias when analyzing the outcomes, they reported that the results of the surveys vary in various populations. COVID-19 is a newly diagnosed disease with more unexplained findings that need more evaluation [ 17 ]. Costa *et al*. [ 18 ] examined a large group of children with rhinovirus diseases and found that more than 50% of them had coinfection, mostly with RSV with more severe symptoms. All cases had a severe respiratory disorder and the diagnosis of pneumonia was common [ 18 ].

Semple *et al*. [ 19 ] discovered a powerful correlation between the coinfection of human metapneumovirus and RSV in newborns with bronchiolitis and dual infection presented a ten-fold increase in relative risk of admittance to a pediatric intensive-care unit. The current research focused on the co-infection of SARS-COV-2 and RSV in older communities. Coinfection of RSV had a seasonality that exactly matches up with the severity of the disease [ 19 ]. There are limited researches regarding viral superinfection with COVID-19 in the literature. Therefore, comparing and analyzing the findings are so complicated and need a long time and more effort.

Kumar *et al*. [ 20 ] presented a patient with a coinfection of tuberculosis and SARS-COV-2 with serious systemic symptoms and an absence of radiological findings detailed to TB. This is a major warning for the Iranian population that TB has increased in many parts of our country [ 20 ].

Langford *et al*. [ 21 ] determined the frequency of bacterial co-infection (at presentation) and secondary infection (after presentation) in the cases with COVID-19. They found that bacterial co-infection (estimated on presentation) was detected in 3.5% of subjects and secondary bacterial infection in 14.3% of the studied population. The overall percentage of SARS-COV-2 infected subjects with bacterial contamination was 6.9%. The infection was more frequent in seriously ill cases [ 21 ].

Because the symptoms of coronavirus infection are similar to those of other flulike disorders, affected patients were tested for RSV in the early weeks of the disease. Early articles from China indicated that coinfection with other respiratory viruses was uncommon [ 22 ].

Coinfected cases did not differ prominently in age from those contaminated with SARS-CoV-2. We evaluated the young adults, adults, and elderly cases, and we found a higher rate of co-infection in the corona virus group. Further studies on the larger communities and all age groups are necessary for more accurate results.

 PCR method for the detection of respiratory disease showed that there is practically concurrent virus infections were seen in patients positive for SARS-CoV-2, but moreover, with the spread of SARS-CoV-2, other respiratory pathogens have seemed to fade away. 

Calcagno *et al*. [ 23 ] examined the nasopharyngeal swab, bronchoalveolar lavage, bronchoaspirate, and sputum of adult and elderly cases with COVID-19 and found RSV infection in 2 cases of 56 infected subjects [ 23 ].

Kim *et al*. [ 12 ] study demonstrated that among 116 SARS-CoV-2 studied cases, 5.2% were infected with RSV, which is lower than our report [ 12 ]. This finding is not in accordance with us, the difference may be associated with the seasonal variation of the virus and the personal characteristic, immune response baseline, and ethnicity of the subjects.

The study of Pinky and Dobrovolny [ 14 ] indicated that in the course of coinfection, one virus can block another easily by being the earliest to contaminate the existing host cells; there is no necessity for biological interfering through immune response interfaces.

According to Lin *et al*. [ 24 ], viral infections of respiratory diseases are common in COVID-19 infection. They detected at least two types of virus co-infection in 2.2% of 186 cases they examined. The common respiratory viruses were RSV, human respiratory virus (HRV), metapnemo virus (MPV), parainfluenza virus type 2 (PIV2), and coronavirus [ 24 ].

A study of Richardson *et al*. [ 25 ] on COVID-19 cases also showed that entero/rhinovirus, non-SARS-CoV-2 coronavirus, RSV, parainfluenza 3, chlamydia pneumonia, MPV, influenza A, and mycoplasma pneumonia are the most common co-infected viruses [ 25 ].

This study has focused on RSV, however, further studies including other viruses are recommended. Current research yielded a higher percentage of RSV coinfection compared to the findings of Lin *et al*. [ 24 ] and Richardson *et al*. [ 25 ].

According to Li *et al*. [ 26 ], co-infection may significantly inhibit the host immune system and alter the defensive immunological response, and is linked to disease severity. Because the majority of our patients were advanced cases in our research, determining the impact of RSV was difficult. WHO suggested a complete screening test for co-infection possibility in COVID-19 infected cases. Unfortunately, we only evaluated RSV as an RNA virus due to financial restriction and the absence of suitable kits. This study indicated higher rates of co-infection between SARS-CoV-2 and RSV than previously reported [ 24 - 25 ], with no available difference in clinical symptoms in patients with and without this co pathogen.

One of the powers of our survey is that we evaluated viruses in pairs, but the total of infected cases to assess was not significant enough to permit us to make ideal decisions. To the best of our knowledge, our report has a larger but non-appropriate number of cases in pairs of viruses up to now.

To complete, the potential co-infection of RSV in COVID-19 patients with clinical characteristics and patterns of the corona should always be regarded. Moreover, assessing more signs and symptoms should be considered.

Our samples were limited to one location (Shiraz). On the other hand, our country's restrictions also affect the availability of kits, and proper examination tests, lack of proper protective devices, and multiply test types of equipment. On the other hand, no available data was about the prevalence of RSV in Shiraz and data of COVID 19 epidemiology in Iran. However, these results indicate that standard testing for non–SARS-CoV-2 respiratory pathogens in the course of the COVID-19 pandemic is questionable to offer clinical assistance unless a definite cause would alter disease management.

## Conclusion

The outcome of present research showed that concurrent RSV with COVID 19 infection might be seen in the hospitalized patients in Shiraz Southwest of Iran. Further investigations on the larger populations, on other pathogens, in multiple locations of the country, and assessment of the severity of symptoms are indispensable for more accurate results.

## Acknowledgments

The authors would like to thank the Vice-Chancellery of research, Shiraz University of Medical Sciences for supporting this research (Grant #21366). This article is based on the thesis by Sina Masoudy. The authors of would also like to thank Dental Research Development Center, for help in statistical analyses. The demographic data used to support the findings of this study were supplied by the Dentistry School of Shiraz University of Medical Sciences under license and so cannot be made freely available.

## Conflict of Interest

The authors declare that they have no conflict of interest.
